# Supplementary material for: The interactome of intact mitochondria by cross-linking mass spectrometry provides evidence for coexisting respiratory supercomplexes
Source: Mol Cell Proteomics. 2017 Dec 8;17(2):216–32. doi: 10.1074/mcp.RA117.000470 (PMC5795388; doi:10.1074/mcp.RA117.000470)
Supplement: Supplemental Data [file supp_RA117.000470_133922_0_supp_23978_fzffwf.zip › spectra_annotation/mito_spectra_annotation/220-1-5-1-2-1.pdf]

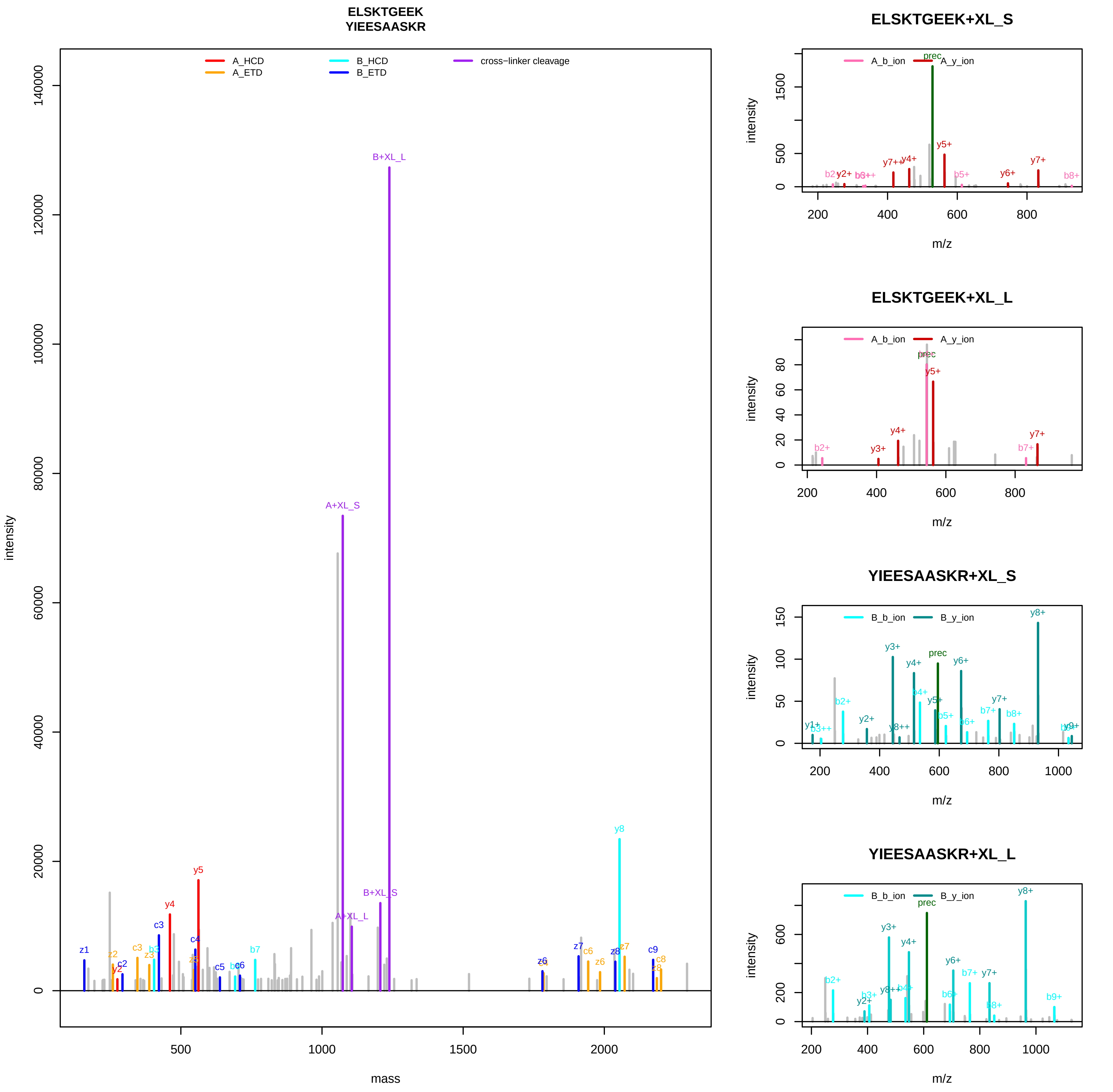
ELSKTGEEK
YIEESAASKR
A_HCD
A_ETD
B_HCD
B_ETD
cross−linker cleavage
B+XL_L
A+XL_S
B+XL_S
A+XL_L
y8
y5
y4
c3
c4
z1
z2
y2
c2
c3
z3
b3
c5
c6
b7
z6
z7
c6
z6
z8
z7
c9
c8
z8
intensity
mass
0
20000
40000
60000
80000
100000
120000
140000
500
1000
1500
2000
ELSKTGEEK+XL_S
ELSKTGEEK+XL_L
A_b_ion
A_y_ion
prec
y5+
y4+
y7++
y7+
y6+
y2+
b2+
b5+
b8+
y3+
b7+
YIEESAASKR+XL_S
YIEESAASKR+XL_L
B_b_ion
B_y_ion
y8+
y3+
y4+
y6+
y7+
y5+
b4+
b5+
b6+
b7+
b8+
b2+
y2+
y8++
y1+
b3++
b9+
y9+
b3+
m/z
intensity
